# Multi-Sequence MR-Based Radiomics Signature for Predicting Early Recurrence in Solitary Hepatocellular Carcinoma ≤5 cm

**DOI:** 10.3389/fonc.2022.899404

**Published:** 2022-06-08

**Authors:** Leyao Wang, Xiaohong Ma, Bing Feng, Shuang Wang, Meng Liang, Dengfeng Li, Sicong Wang, Xinming Zhao

**Affiliations:** ^1^ Department of Diagnostic Radiology, National Cancer Center/National Clinical Research Center for Cancer/Cancer Hospital, Chinese Academy of Medical Sciences and Peking Union Medical College, Beijing, China; ^2^ Magnetic Resonance Imaging Research, General Electric Healthcare, Beijing, China

**Keywords:** magnetic resonance imaging, early recurrence, radiomics, nomogram, hepatocellular carcinoma

## Abstract

**Purpose:**

To investigate the value of radiomics features derived from preoperative multi-sequence MR images for predicting early recurrence (ER) in patients with solitary hepatocellular carcinoma (HCC) ≤5 cm.

**Methods:**

One hundred and ninety HCC patients were enrolled and allocated to training and validation sets (n = 133:57). The clinical–radiological model was established by significant clinical risk characteristics and qualitative imaging features. The radiomics model was constructed using the least absolute shrinkage and selection operator (LASSO) logistic regression algorithm in the training set. The combined model was formed by integrating the clinical–radiological risk factors and selected radiomics features. The predictive performance was assessed by the area under the receiver operating characteristic curve (AUC).

**Results:**

Arterial peritumoral hyperenhancement, non-smooth tumor margin, satellite nodules, cirrhosis, serosal invasion, and albumin showed a significant correlation with ER. The AUC of the clinical–radiological model was 0.77 (95% CI: 0.69–0.85) and 0.76 (95% CI: 0.64–0.88) in the training and validation sets, respectively. The radiomics model constructed using 12 radiomics features selected by LASSO regression had an AUC of 0.85 (95% CI: 0.79–0.91) and 0.84 (95% CI: 0.73–0.95) in the training and validation sets, respectively. The combined model further improved the prediction performance compared with the clinical–radiological model, increasing AUC to 0.90 (95% CI: 0.85–0.95) in the training set and 0.88 (95% CI: 0.80–0.97) in the validation set (p < 0.001 and p = 0.012, respectively). The calibration curve fits well with the standard curve.

**Conclusions:**

The predictive model incorporated the clinical–radiological risk factors and radiomics features that could adequately predict the individualized ER risk in patients with solitary HCC ≤5 cm.

## Introduction

Hepatocellular carcinoma (HCC) is the sixth most common malignancy and the third leading cause of cancer-related mortality globally (1). In China, newly diagnosed cases of HCC account for almost half of the global cases annually, which seriously threatens the life and health of the Chinese people (2). For patients with early-stage HCC (solitary HCC ≤5 cm or up to three nodules ≤3 cm, without macrovascular invasion and extrahepatic spread) and adequate liver function (3), hepatectomy is still widely accepted as the first-line treatment option in most centers; in particular, early solitary HCC is an ideal surgical indication in clinical practice. Unfortunately, the long-term survival in patients with HCC remains unsatisfactory, with the 5-year recurrence rate at 50%–70% (4).

According to the current clinical practice guidelines, HCC recurrence is usually divided into early and late recurrence by the 2-year cutoff point (5–8). Early recurrence (ER) accounts for more than 70% of tumor recurrence, which is likely caused by occult metastasis of the primary tumor (6). The time of recurrence is a significant survival factor, and the overall survival time for HCC patients with ER is often lower than for those without ER (8–10). Previous studies have reported several risk factors of ER, such as large tumor volume, multiple tumors, poor differentiation, satellite lesions, non-smooth tumor margins, vascular invasion, and peritumoral parenchymal enhancement in the arterial phase (AP) (11–16).

Radiomics is a process of converting digital medical images into high-throughput, innumerable quantitative features using different algorithms, which provide valuable diagnostic, prognostic, or predictive information (17). To date, radiomics has been used to predict the postoperative ER of other types of cancer (18–20). As a non-invasive and effective tool, radiomics plays an important role in predicting ER of HCC after hepatectomy, transcatheter arterial chemoembolization, and radiofrequency ablation (15, 16, 21,), with relatively excellent diagnostic accuracy. However, few studies focused on radiomics analysis derived from multi-sequence MR images to predict postoperative ER of solitary HCC with a diameter ≤ 5 cm.

Previous studies showed that tumor diameter greater than 5 cm was closely related to ER and high mortality (22–24). However, few studies specifically predict ER of solitary HCC with a diameter ≤ 5 cm after hepatectomy. Therefore, it is very important to identify risk factors related to ER for guiding further clinical treatment and improving the long-term survival of HCC patients.

The aim of this study was to develop and validate an effective and visualized model based on multi-sequence MR images to predict ER in patients with solitary HCC ≤5 cm.

## Materials and Methods

### Patients

**Figure 1:**
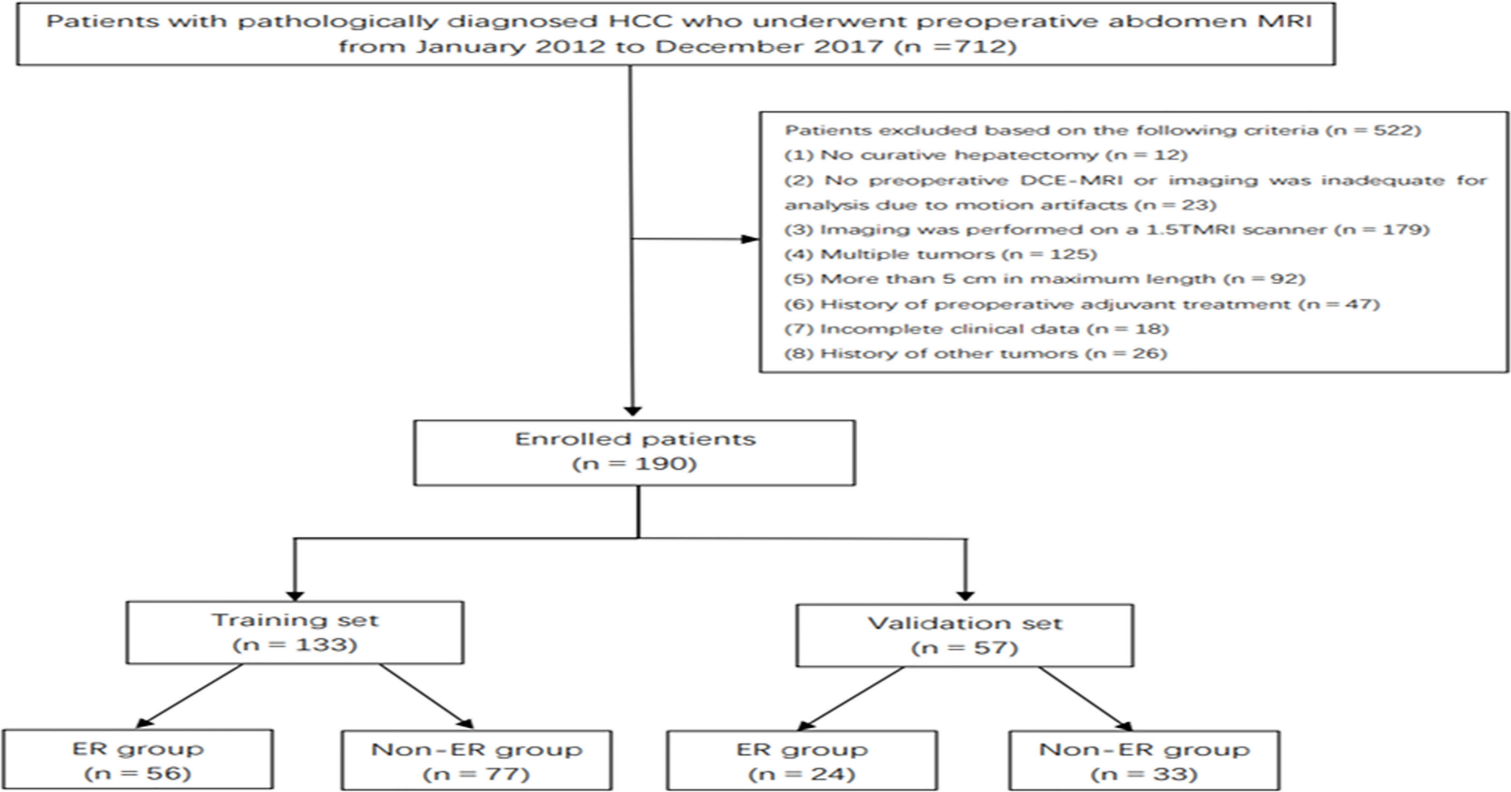
A flowchart of the study cohort.

This retrospective study received ethical approval, and the requirement for informed consent was waived. From January 2012 to December 2017, 712 consecutive patients underwent R0 resection in our hospital. The inclusion criteria were the following: a) histologically proven HCC with a negative resection margin, b) solitary tumor ≤5 cm, c) no preoperative history of cancer-related treatments (including surgery and interventional therapy), d) high-quality MR images performed 4 weeks preoperatively (the lesions were clearly displayed without obvious external and respiratory motion artifacts), and e) at least 2 years of follow-up. Finally, a total of 190 HCC patients (80 patients with ER and 110 patients with non-ER) were included in this retrospective study. The enrolled patients were divided into a training set (56 patients with ER and 77 patients with non-ER) and a validation set (24 patients with ER and 33 patients with non-ER) at a ratio of 7:3 (**Figure 1**).

The clinical and pathological variables were obtained from the electronic medical record system for all patients, including demographic characteristics, preoperative laboratory data, and postoperative pathological data.

### MRI Acquisition and Imaging Analysis

All MR examinations were performed using 3.0 T scanners (Signa HDx, GE Medical System, Milwaukee, WI, USA; Discovery MR 750, GE Medical System) with an 8-channel phased-array body coil. After localizer images were obtained, in-phase and opposed-phase T1-weighted imaging, fat-suppression T2-weighted imaging (T2WI/FS), diffusion-weighted imaging (DWI; b-values of 0 and 800 s/mm^2^), and dynamic contrast-enhanced (DCE) T1-weighted three-dimensional spoiled gradient echo liver acceleration volume acquisition were performed. The contrast-enhanced images were acquired at 20–30 s (AP), 60–70 s (portal venous phase (PVP)), and 180 s (delayed phase (DP)). Gadodiamide (Omniscan 0.5 mmol/ml; GE Healthcare) at a standard dose (0.2 ml/kg) was administered at a rate of 2.0 ml/s and flushed with 20 ml of 0.9% sterile saline *via* an automatic injector.

Two abdominal radiologists (LW and BF with 3 and 6 years’ experience, respectively) reviewed all MR images. Both radiologists were blinded to any clinical and pathological information. They reached a consensus through discussion when any disagreements existed. They independently evaluated and recorded the following basic MR image features: a) maximum tumor diameter (maximum diameter measured on axial MR images in the PVP), b) liver background (cirrhosis or non-cirrhosis), c) location (left lobe, right lobe, left and right lobes, or caudate lobe), d) intratumoral fat (presence or absence, defined as the signal in the opposed-phase reduced compared to the in-phase), e) DWI intensity (hyperintense or slightly hyperintense), f) capsule (complete or absent/incomplete), g) dynamic enhancement pattern (gradual enhancement, persistent enhancement, wash in and wash out, or minimal/no enhancement), h) tumor margin (smooth or non-smooth), and i) arterial peritumoral hyperenhancement (APHE; defined as relatively high intensity of the liver parenchyma outside the tumor boundary in AP that became isointense in the subsequent phases) (12).

### Tumor Segmentation and Radiomics Feature Extraction

T2WI/FS images and three-phase DCE-MR images were used for feature extraction. Before tumor segmentation, all preoperative MR images were resampled into a uniform voxel size of 1 × 1 × 1 mm^3^ using Artificial Intelligence Kit software (version 3.3.0, GE Healthcare, China). Three-dimensional manual segmentation was performed by a radiologist with 3 years’ MR experience using ITK-SNAP software (v.3.6.0;www.itksnap.org;open-source software). The volumes of interest (VOIs) were manually drawn along the boundary of the tumor on each consecutive slice for all 190 lesions. To assess the intraclass correlation coefficient (ICC), 40 VOIs were randomly chosen and performed independently by another radiologist with 6 years’ experience. In total, 1,316 radiomics features were extracted from each sequence using the Artificial Intelligence Kit software based on the open-source Pyradiomics python package, which included the following parameters: first-order histogram features (n = 18), texture features (n = 89, including 14 shape features, 16 gray-level zone size matrix (GLZSM) features, 16 gray-level run-length matrix (RLM) features, 24 gray-level co-occurrence matrix (GLCM) features, 14 gray-level dependence matrix features, and 5 neighboring gray-tone difference matrix features), wavelet features (n = 744), local binary pattern features (n = 279), and Laplacian of Gaussian (logSigma = 2.0/3.0) features (n = 186).

### Radiomics Feature Selection and Signature Construction

**Figure 2:**
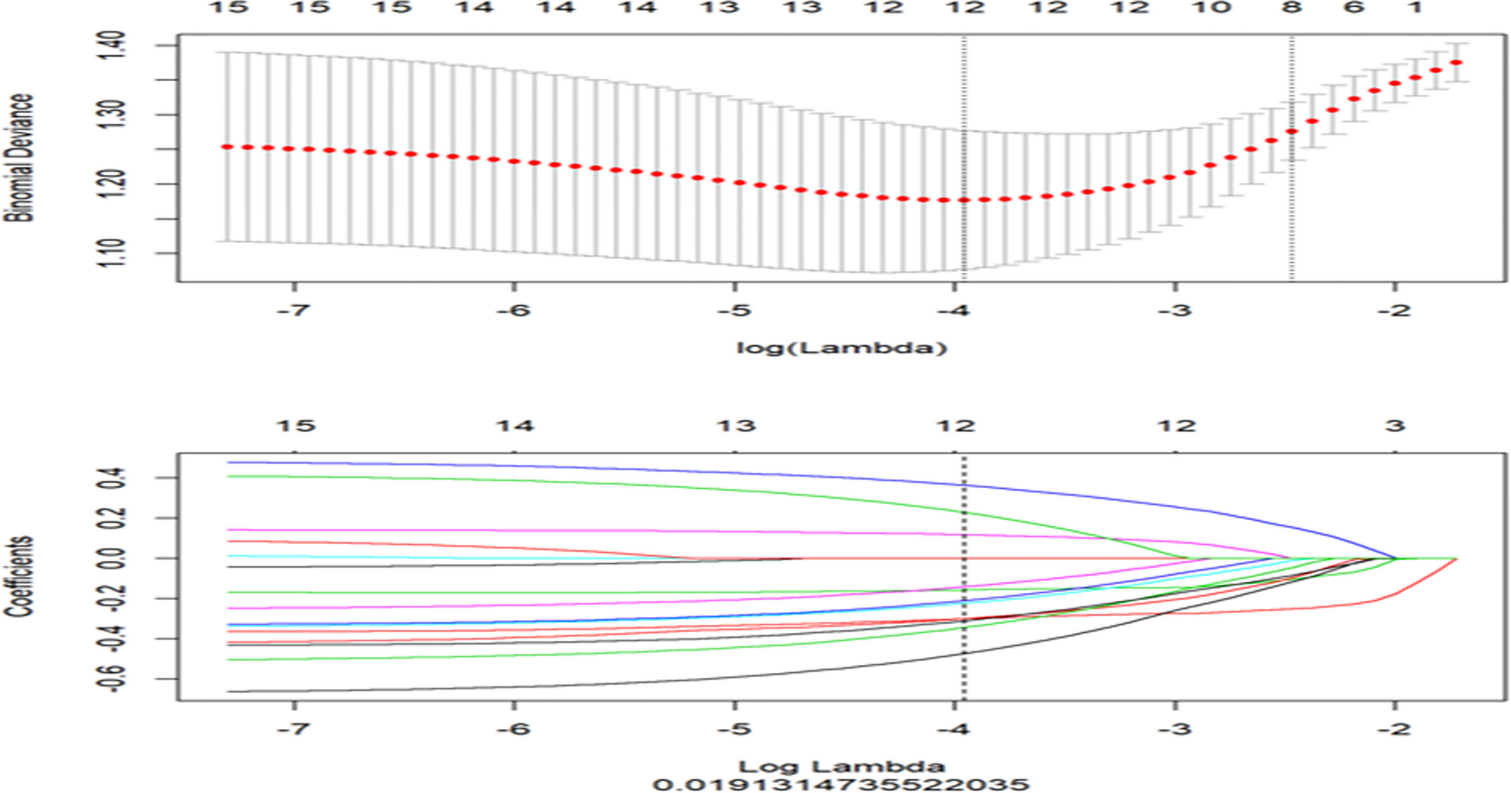
The least absolute shrinkage and selection operator (LASSO) regression for radiomics features selection and signature construction. The top graph represents in LASSO model, with the penalization parameter λ selection using 10-fold cross-validation as the minimum criteria. The log (λ) (x-axis) was plotted against the partial likelihood of deviance (y-axis). Dotted vertical lines were drawn at the minimum criteria and the 1 − SE criteria. λ value of 0.019, with log (λ), −3.96 was chosen (1 − SE criteria). The bottom graph represents LASSO coefficient profiles of the radiomics features. Ten-fold cross-validation in the log (λ) sequence was used to draw the vertical line at the value selected; also indicated are 12 features with non-zero coefficients.

Features with ICC > 0.75 indicated satisfactory consistency and were retained for subsequent analysis. The least absolute shrinkage and selection operator (LASSO) logistic regression algorithm was used to identify the most predictive radiomics features, and 10-fold cross validation was used to tune the model parameter as the inner resampling loop (**Figure 2**). The radiomics score (Rad-score) was calculated *via* the linear combination of the selected features weighted by their respective LASSO coefficients. Considering the small sample size of our datasets, this radiomics model was further verified by using 100-time bootstrap for the outer resampling loop. The whole dataset was randomly divided into the training set and validation set 100 times. The existing radiomics model was tested on the new 100 testing datasets.

Clinical–radiological variables with p < 0.05 in the univariate analysis were included in the multivariate logistic regression analysis to confirm risk factors associated with ER, and the clinical–radiological model was generated. A combined model was developed by incorporating the clinical–radiological risk factors and the Rad-score. Receiver operating characteristic (ROC) curves were generated for those three models (a clinical–radiological model, a radiomics model, and a combined model). Accuracy, sensitivity, specificity, positive predictive value (PPV), negative predictive value (NPV), and the area under the ROC curve (AUC) were calculated.

### Follow-Up

Serum alpha-fetoprotein (AFP) levels and contrast-enhanced CT/MRI were performed every 3–6 months for 2 years after surgery. ER was defined as intrahepatic tumor relapse (with typical HCC imaging features or confirmed by pathology) and metastasis (distant metastasis or lymph node metastases) within 2 years after surgery.

### Statistical Analysis

Categorical variables were compared by using the chi-square test or Fisher’s exact test, and continuous variables were compared by using Student’s t-test or the Mann–Whitney U test, as appropriate. All statistical analyses were performed with SPSS software (version 25.0, IBM). The performance of each model was compared using the Delong test. A two-sided p < 0.05 indicated a statistical significance.

## Results

### Clinical Characteristics

Overall, 190 HCC patients (male/female, 163/27; mean age, 54.86 ± 9.03 years; age range, 27–80 years) who met the inclusion criteria were included and divided into the training set (n = 133; male/female, 112/21; mean age, 54.39 ± 9.04 years) and validation set (n = 57; male/female, 51/6; mean age, 55.95 ± 8.98 years). Eighty (42.1%) of 190 patients with solitary HCC ≤5 cm experienced postoperative ER. Of the 80 patients with ER, cirrhosis was presented in 63 patients, and cirrhosis was strongly associated with ER in both the training set (p = 0.011) and validation set (p = 0.001). Except for prothrombin time (p = 0.047), no statistical difference was observed between the two sets in the clinical and radiological characteristics (all p > 0.05), as shown in **Tables 1a**, **1b**.

**Table 1a:** Comparisons of clinical factors in the training and validation sets.

Characteristic	Training set (N = 133)			Validation set (N = 57)			P_inter_
	ER (N = 56)	Non-ER (N = 77)	P_intra_	ER (N = 24)	Non-ER (N = 33)	P_intra_	
Age (years), mean ± SD	56.32 ± 10.08	52.99 ± 7.98	0.035	56.92 ± 9.51	55.24 ± 8.65	0.492	0.277
Gender (male/female)	47/9	65/12	0.939	19/5	32/1	0.084	0.341
Hepatitis, no. (%)			0.189			0.013	0.922
Hepatitis B	40 (71.43%)	63 (81.82%)		19 (79.17%)	20 (60.61%)		
Hepatitis C	5 (8.93%)	2 (2.60%)		4 (16.67%)	2 (6.06%)		
None	11 (19.64%)	12 (15.58%)		1 (4.17%)	11 (33.33%)		
AFP level (U/ml), no. (%)			0.846			0.972	0.652
<400	43 (76.79%)	58 (75.32%)		19 (79.17%)	26 (78.79%)		
≥400	13 (23.21%)	19 (24.68%)		5 (20.83%)	7 (21.21%)		
Satellite lesions, no. (%)			0.002			0.847	0.37
Present	12 (21.43%)	3 (3.90%)		2 (8.33%)	2 (6.06%)		
Absent	44 (78.57%)	74 (96.10%)		22 (91.67%)	31 (93.94%)		
Histologic grade, no. (%)			0.808			0.448	0.308
Well	4 (7.14%)	8 (10.39%)		1 (4.17%)	1 (3.03%)		
Moderate	35 (62.50%)	47 (61.04%)		18 (75.00%)	20 (60.61%)		
Poor	17 (30.36%)	22 (28.57%)		5 (20.83%)	12 (36.36%)		
T stage, no. (%)			0.018			0.481	0.844
I	35 (62.50%)	64 (83.12%)		16 (66.67%)	24 (72.73%)		
II	18 (32.14%)	12 (15.58%)		7 (29.17%)	9 (27.27%)		
III	2 (3.57%)	1 (1.30%)		1 (4.17%)	0 (0.00%)		
IV	1 (1.79%)	0 (0.00%)		0	0		
MVI, no. (%)			0.01			0.926	0.891
Present	24 (42.86%)	17 (22.08%)		7 (29.17%)	10 (30.30%)		
Absent	32 (57.14%)	60 (77.92%)		17 (70.83%)	23 (69.70%)		
Serosal invasion			0.016			0.516	0.409
Present	38 (67.86%)	36 (46.75%)		13 (54.17%)	15 (45.45%)		
Absent	18 (32.14%)	41 (53.25%)		11 (45.83%)	18 (54.55%)		
ALT (U/L)	27.00 (17.45, 38.55)	26.00 (18.00, 34.30)	0.92	32.50 (21.90, 72.55)	19.00 (16.70, 32.00)	0.001	0.839
AST (U/L)	28.00 (20.45, 35.00)	24.00 (20.00, 30.00)	0.152	29.50 (24.00, 41.10)	22.00 (18.00, 27.30)	0.001	0.968
LDH (U/L)	167.50 (151.70, 185.55)	163.00 (145.70, 182.30)	0.232	171.00 (148.75, 189.50)	172.00 (145.50, 186.50)	0.679	0.841
GGT (U/L)	44.00 (27.00, 68.10)	34.00 (21.00, 53.60)	0.041	52.00 (26.45, 84.20)	29.00 (20.50, 42.90)	0.024	0.81
TBIL (μmol/L)	73.50 (61.00, 86.00)	12.30 (8.74, 15.92)	0.929	11.55 (9.05, 13.60)	13.60 (11.50, 16.35)	0.097	0.596
DBIL (μmol/L)	4.60 (3.49, 6.46)	4.40 (3.17, 5.83)	0.443	4.55 (3.84, 5.61)	4.60 (3.77, 6.33)	0.948	0.58
IBIL (μmol/L)	6.90 (5.33, 9.25)	7.50 (5.67, 10.00)	0.402	4.55 (3.84, 5.61)	4.60 (3.77, 6.33)	0.948	0.586
TP (g/L)	70.10 (63.60, 75.58)	71.20 (67.60, 75.75)	0.252	68.80 (66.33, 72.98)	70.40 (67.80, 75.45)	0.386	0.499
ALB (g/L)	42.45 (39.30, 44.98)	44.10 (40.90, 46.90)	0.02	40.95 (38.73, 42.97)	44.70 (41.97, 47.93)	<0.001	0.684
G (g/L)	26.65 (23.80, 30.41)	26.80 (24.24, 29.23)	0.765	28.05 (25.48, 29.80)	25.30 (22.80, 28.25)	0.028	0.953
PLT (10 * 9/L)	156.50 (126.00, 203.65)	165.00 (125.70, 199.30)	0.947	153.00 (114.50, 179.75)	155.00 (112.50, 195.50)	0.794	0.124
PT (s)	11.80 (11.20, 12.38)	11.60 (11.20, 12.40)	0.815	11.50 (10.85, 12.27)	11.20 (10.77, 12.06)	0.599	0.047

**Table 1b:** Comparisons of radiological features in the training and validation sets.

Characteristic	Training set (N = 133)			Validation set (N = 57)			P_inter_
	ER (N = 56)	non-ER (N = 77)	P_intra_	ER (N = 24)	non-ER (N = 33)	P_intra_	
Tumor size (cm), mean (range)	3.15 (2.70, 4.10)	3.00 (2.27, 4.00)	0.219	3.60 (2.95, 4.50)	3.10 (2.30, 4.05)	0.089	0.288
Cirrhosis, no. (%)			0.011			0.001	0.922
Present	42 (75.00%)	41 (53.25%)		21 (87.50%)	15 (45.45%)		
Absent	14 (25.00%)	36 (46.75%)		3 (12.50%)	18 (54.55%)		
Intratumoral fat, no. (%)			0.234			0.838	0.664
Present	6 (10.71%)	14 (18.18%)		5 (20.83%)	5 (15.15%)		
Absent	50 (89.29%)	63 (81.82%)		19 (79.17%)	28 (84.85%)		
Lesion location, no. (%)			0.337			0.085	0.181
Left lobe	8 (14.29%)	20 (25.97%)		2 (8.33%)	7 (21.21%)		
Right lobe	44 (78.57%)	53 (68.83%)		22 (91.67%)	22 (66.67%)		
Left and right lobes	1 (1.79%)	2 (2.60%)		0 (0.00%)	4 (12.12%)		
Caudate lobe	3 (5.36%)	2 (2.60%)		0 (0.00%)	0 (0.00%)		
Shape, no. (%)			0.753			0.426	0.727
Regular	29 (51.79%)	42 (54.55%)		12 (50.00%)	20 (60.61%)		
Irregular	27 (48.21%)	35 (45.45%)		12 (50.00%)	13 (39.39%)		
Radiological capsule			0.753			0.93	0.498
Complete	32 (57.14%)	45 (58.44%)		15 (62.50%)	21 (63.64%)		
Absence or incomplete	24 (42.86%)	32 (41.56%)		9 (37.50%)	12 (36.36%)		
Lesion margin, no. (%)			0.007			0.929	0.352
Smooth	35 (62.50%)	64 (83.12%)		20 (83.33%)	26 (78.79%)		
Non-smooth	21 (37.50%)	13 (16.88%)		4 (16.67%)	7 (21.21%)		
DWI intensity, no. (%)			0.494			0.919	0.79
Hyperintense	47 (83.93%)	65 (84.42%)		21 (87.50%)	28 (84.85%)		
Slightly hyperintense	9 (16.07%)	12 (15.58%)		3 (12.50%)	5 (15.15%)		
Enhancement pattern, no. (%)			0.046			0.181	0.432
Wash in and wash out	42 (75.00%)	57 (74.03%)		22 (91.67%)	24 (72.73%)		
Gradual enhancement	4 (7.14%)	0 (0.00%)		0 (0.00%)	0 (0.00%)		
Persistent enhancement	3 (5.36%)	11 (14.29%)		1 (4.17%)	2 (6.06%)		
No or minimal enhancement	7 (12.50%)	9 (11.69%)		1 (4.17%)	7 (21.21%)		
Arterial peritumoral enhancement, no. (%)			0.037			0.204	0.606
Present	9 (16.07%)	4 (5.19%)		5 (20.83%)	2 (6.06%)		
Absent	47 (83.93%)	73 (94.81%)		19 (79.17%)	31 (93.94%)		

P_Intra_ indicates whether significant differences exist between the two groups. P_Inter_ represents whether significant differences exist between the two sets.

AFP, alpha-fetoprotein; ALT, alanine transaminase; AST, aspartate aminotransferase; LDH, lactate dehydrogenase; GGT, gamma-glutamyl transpeptidase; TBIL, total bilirubin; DBIL, direct bilirubin; IBIL, indirect bilirubin; TP, total protein; ALB, albumin; G, globulin; PLT, platelets; PT, prothrombin time; MVI, microvascular invasion; ER, early recurrence; IQR, interquartile range.

### Clinical–Radiological Model Construction and Validation

Univariate analysis showed that eleven clinical and radiological characteristics including age, cirrhosis, enhancement pattern, non-smooth tumor margin, APHE, T stage, microvascular invasion (MVI), satellite nodules, serosal invasion, gamma-glutamyl transpeptidase level, and albumin level were significantly different between the ER and non-ER groups in the training set (all p < 0.05). Multivariate logistic regression analysis demonstrated that the APHE (odds ratio [OR], 5.03; 95% CI, 1.18–21.43), non-smooth tumor margin (OR, 0.42; 95% CI, 0.16–1.05), satellite nodules (OR, 6.21; 95% CI, 1.45–26.62), cirrhosis (OR, 2.98; 95% CI, 1.20–7.39), serosal invasion (OR, 2.08; 95% CI, 0.91–4.73), and albumin (OR, 0.89; 95% CI, 0.80–0.99) were independent predictors for ER in the training set, which were used to construct the clinical–radiological model (**Table 2**). The AUCs of the clinical–radiological model were 0.77 (95% CI: 0.69–0.85) in the training set and 0.76 (95% CI: 0.64–0.88) in the validation set.

**Table 2:** Univariate and multivariate analyses for early recurrence in the training set.

Variables	Univariate analysis		Multivariate analysis	
	Odds ratio (95% CI)	p-Value	Odds ratio (95% CI)	p-Value
Age	1.043 [1.002–1.086]	0.035	–	0.243
Cirrhosis	2.634 [1.241–5.590]	0.011	2.977 [1.200–7.388]	0.019
Enhancement pattern	0.847 [0.605–1.186]	0.046		0.582
Non-smooth tumor margin	0.339 [0.151–0.757]	0.007	0.416 [0.164–1.054]	0.064
Arterial peritumoral enhancement	3.495 [1.018–11.998]	0.037	5.029 [1.180–21.434]	0.029
T stage	2.571 [1.268–5.211]	0.018	–	0.760
Microvascular invasion	2.647 [1.244–5.632]	0.01	–	0.296
Satellite nodules	6.717 [1.799–25.125]	0.002	6.209 [1.448–26.621]	0.014
Serosal invasion	2.404 [1.173–4.928]	0.016	2.076 [0.912–4.726]	0.082
Gamma-glutamyl transpeptidase (U/L)	1.003 [0.998–1.008]	0.041	–	0.275
Albumin (g/L)	0.898 [0.819–0.985]	0.02	0.889 [0.789–0.990]	0.032

### Radiomics Model Construction and Validation

Among 1,316 radiomics features extracted from multi-sequence MR images, the LASSO analysis selected 12 features with non-zero coefficients to calculate the Rad-score (two, two, two, and six features from T2WI, AP, PVP, and DP images). The following formula was used to obtain the corresponding Rad-score for each patient: Rad-score=-0.3*T2_original_shape_Sphericity-0.157*AP_wavelet_HLL_glcm_ClusterShade+0.363*DP_lbp_3D_k_glrlm_ShortRunLowGrayLevelEmphasis-0.223*AP_wavelet_LHH_glszm_HighGrayLevelZoneEmphasis+0.118*DP_wavelet_LHL_glcm_ClusterProminence-0.473*DP_wavelet_LLL_firstorder_Minimum-0.3*VP_original_glrlm_LongRunLowGrayLevelEmphasis-0.343*T2_log_sigma_2_0_mm_3D_glrlm_LongRunEmphasis-0.21*DP_wavelet_LHL_firstorder_Skewness-0.141*DP_lbp_3D_k_firstorder_10Percentile-0.31*VP_original_shape_Sphericity+0.228*DP_log_sigma_3_0_mm_3D_glcm_ClusterProminence-0.411.

The AUCs of the radiomics model were 0.85 (95% CI: 0.79–0.91) in the training set and 0.84 (95% CI: 0.73–0.95) in the validation set. The outer resampling loop using the 100-time bootstrap method delivered a mean AUC of 0.85 (range from 0.70 to 0.96). Among them, 86% of AUC values were greater than 0.80, which showed good reliability of this radiomics model.

### Combined Model Construction and Validation

The combined model was developed by incorporating the clinical–radiological risk factors and the Rad-score. The AUCs of the combined model were 0.90 (95% CI, 0.85–0.95) in the training set and 0.88 (95% CI, 0.80–0.97) in the validation set. In the training set, the combined model displayed accuracy, sensitivity, specificity, PPV, and NPV of 81.20%, 71.83%, 91.94%, 91.01%, and 74.03%, respectively. When applied in the validation set, the combined model exhibited accuracy, sensitivity, specificity, PPV, and NPV of 84.21%, 85.71%, 83.33%, 75.00%, and 90.91%, respectively. The predictive performances of the clinical–radiological model, radiomics model, and the combined model in the training and validation sets are listed in **Table 3**.

**Table 3:** Predictive performance of the three models.

Model	Training set (N = 133)						Validation set (N = 57)					
	Accuracy	Sensitivity	Specificity	PPV	NPV	AUC (95% CI)	Accuracy	Sensitivity	Specificity	PPV	NPV	AUC (95% CI)
Radiomics model	75.18	80.36	71.43	67.16	83.33	0.85 (0.79–0.91)	71.93	87.50	60.61	61.76	86.96	0.84 (0.73–0.95)
Clinical–radiological model	75.19	81.08	72.92	53.57	90.91	0.77 (0.69–0.85)	66.67	63.16	68.42	50.00	78.79	0.76 (0.64–0.88)
Combined model	81.20	71.83	91.94	91.07	74.03	0.90 (0.85–0.95)	84.21	85.71	83.33	75.00	90.91	0.88 (0.80–0.97)

PPV, positive predictive value; NPV, negative predictive value; AUC, area under the curve.

**Figure 3:**
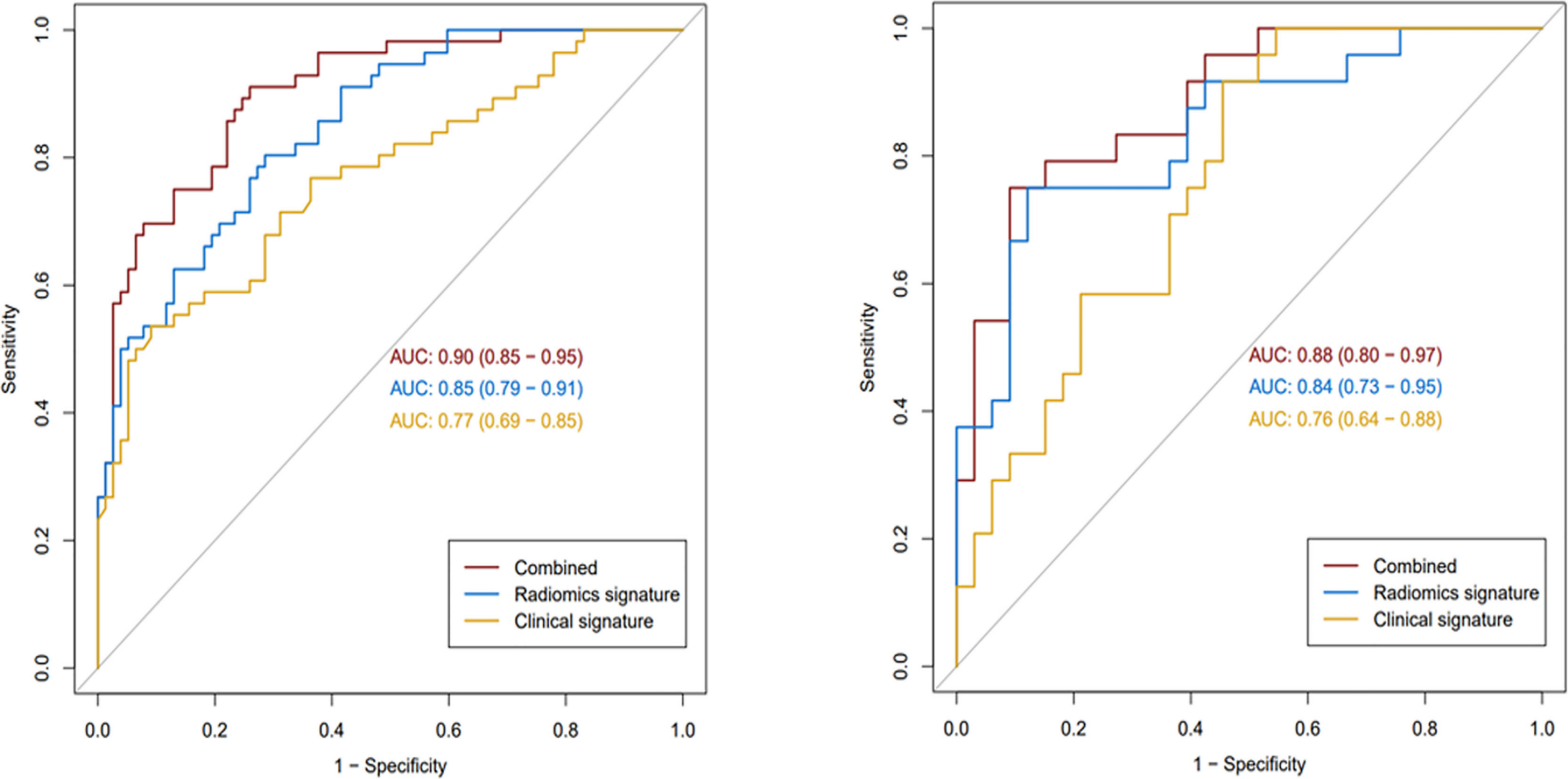
The receiver operating characteristic curves of the three models in the training (left) and validation (right) sets.

For ER prediction, the combined model outperformed both the clinical–radiological model (p < 0.001) and the radiomics model (p = 0.023) in the training set. However, no significant difference was observed between the combined model and the radiomics model in the validation set (p = 0.174), although the combined model showed better performance than the clinical–radiological model in the validation set (p = 0.012). ROC curves for the prediction of ER were compared among the clinical–radiological, radiomics, and combined models (**Figure 3**).

### Nomogram Construction and Validation

**Figure 4:**
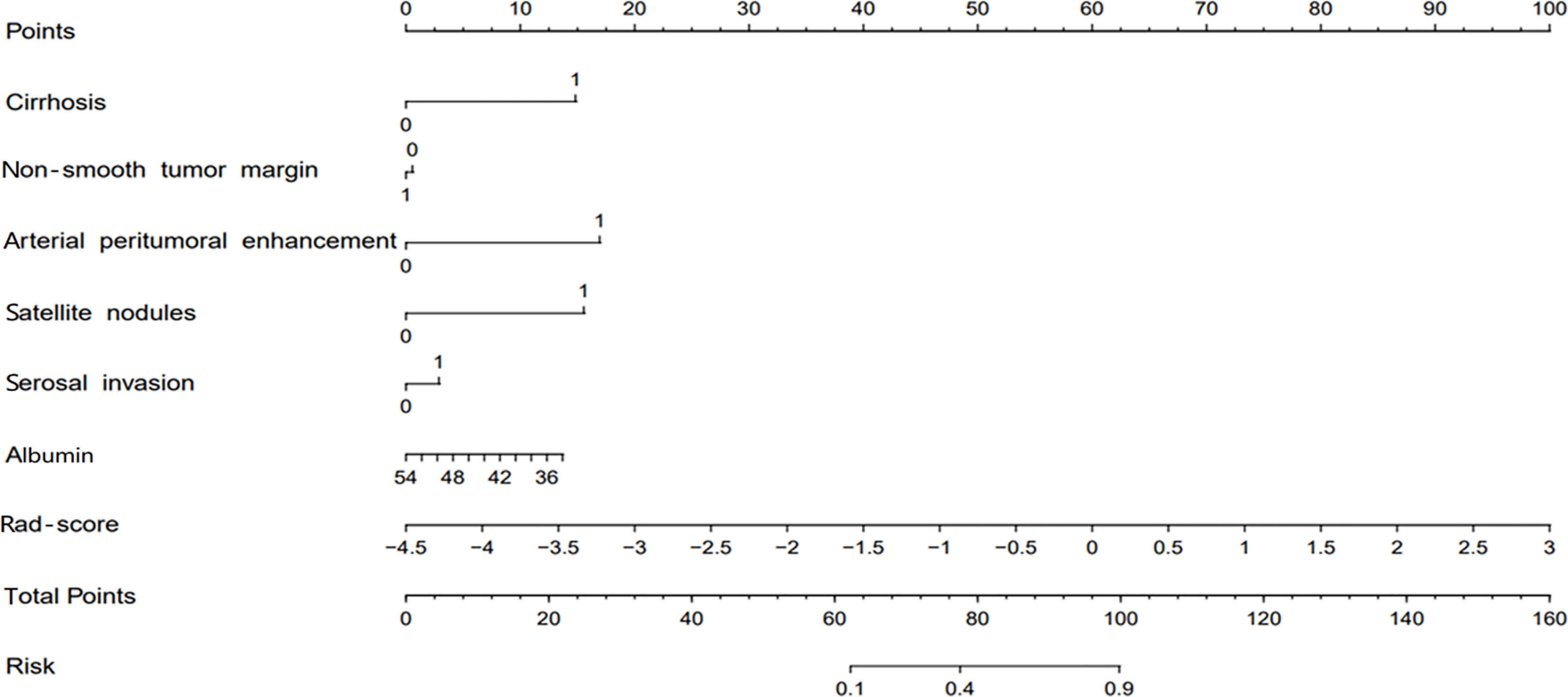
The radiomics nomogram for predicting early recurrence.

**Figure 5:**
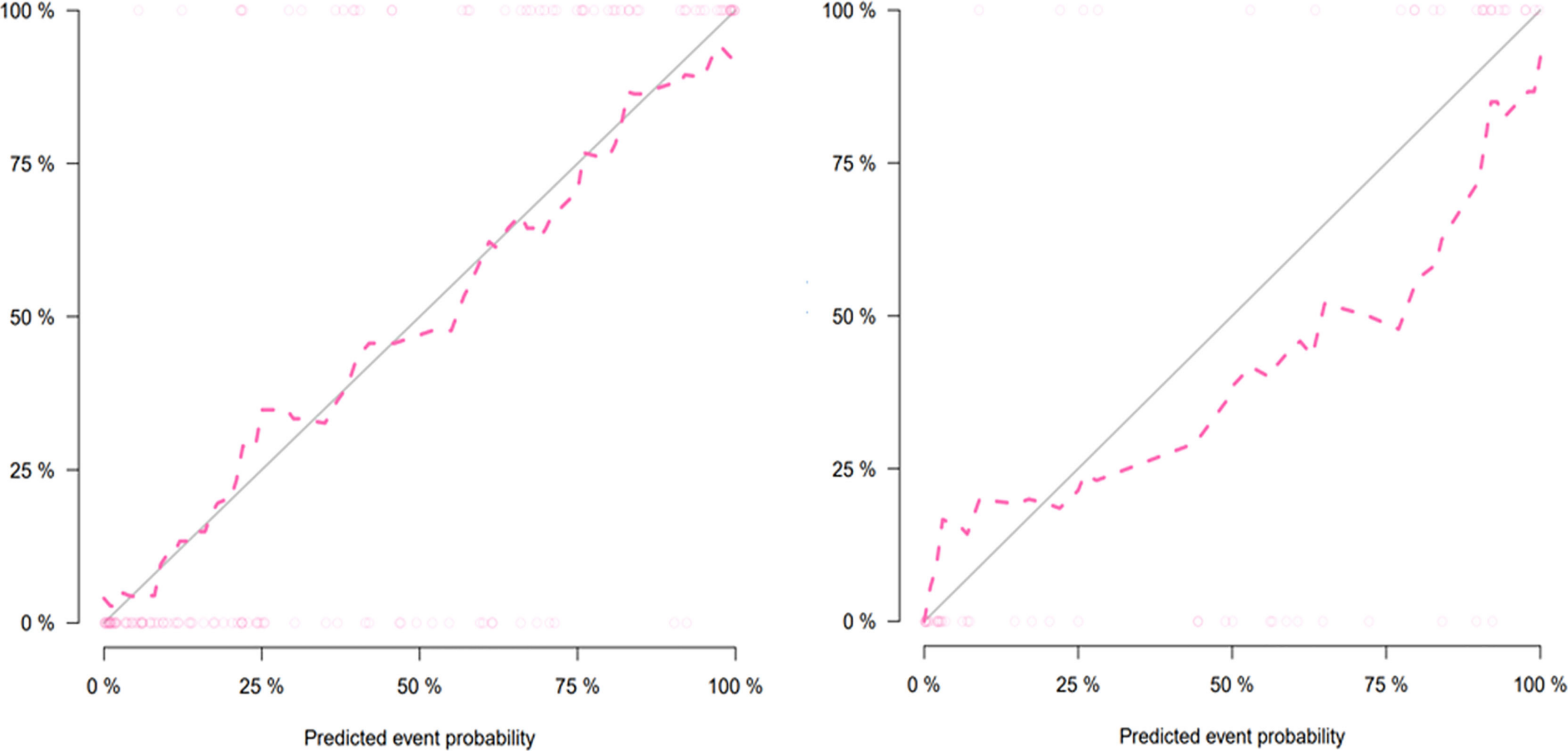
Calibration curves of the nomogram for the training (left) and validation (right) sets. The y-axis and the x-axis show the actual rate of early recurrence (ER) and the predicted ER possibility, respectively. The solid diagonal line represents a perfect prediction. The closer the pink dashed line fits the solid line, the better the predictive ability of the model is.

**Figure 6:**
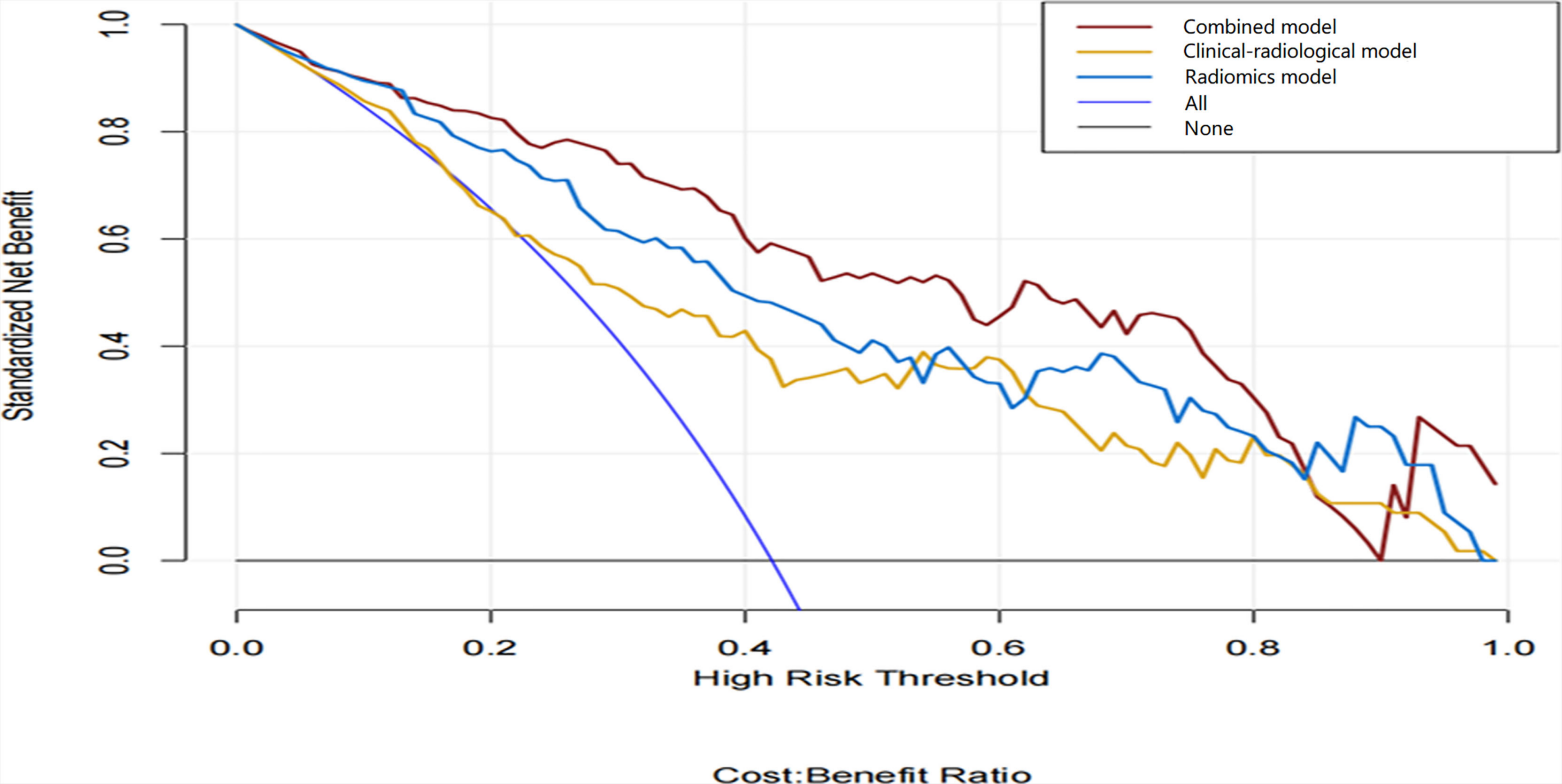
Decision curve analysis of the three models. The y-axis and the x-axis show the standardized net benefit and the threshold probability, respectively. Among the three models, the combined model (red line) has a higher net benefit than the clinical–radiological model (yellow line) and the radiomics model (blue line) within a wide range of threshold probabilities.

The combined model-based nomogram is presented in **Figure 4**. The Hosmer–Lemeshow test yielded no significant difference in both the training and validation sets (all p > 0.05). The calibration curves (**Figure 5**) revealed that the predictive probability of the nomogram was consistent with the actual ER probability in both sets. The decision curve (**Figure 6**) showed that the combined model had the highest net benefit as compared to the clinical–radiological model and the radiomics model within all reasonable threshold probabilities.

## Discussion

In this study, we developed and validated a radiomics-based model to predict ER of HCC patients with solitary tumor ≤5 cm by incorporating clinical–radiological variables and radiomics features extracted from multi-sequence MR images. The combined model achieved satisfactory predictive performance and further improved the prediction performance compared with the clinical–radiological model. The combined nomogram can help the clinical doctors to identify patients at high risk of ER after R0 resection and may provide HCC patients with adequate treatment opportunities and improve their overall survival.

As an emerging quantitative analysis method, radiomics plays an important role in predicting ER of HCC after hepatectomy. However, as far as we know, there were few studies to investigate the relationship between radiomics characteristics based on multi-sequence MR images and ER of single HCC ≤ 5 cm. Zhao et al. (25) found that the radiomics model based on multi-sequence MR images presents the best predictive ability compared with single sequence and other different sequence combinations. Additionally, Zhang et al. (16) developed and validated a radiomics nomogram for predicting ER using whole-lesion radiomics features extracted from multi-sequence MR images. Their results indicated that the radiomics nomogram had a fairly good discriminative performance. Also, our result was consistent with the previous studies. Among all the features of the radiomics model, there were 2 features from T2WI and 10 features from DCE images that indicated that DCE images have more influence on the differential diagnosis of ER. The present study confirmed that the radiomics model based on the preoperative multi-sequence MR images (including T2WI/FS and DCE-MR images) had a higher predictive ability for ER than the clinical–radiological model with AUC values of 0.85 and 0.84 in the training and validation sets, respectively. This result indicated that radiomics features extracted from multi-sequence MR images might contain more biological and heterogeneity information than the clinical–radiological characteristics, which could further improve the predictive performance.

APHE is an auxiliary diagnostic feature of malignant tumors in the liver imaging reporting and data system. Previous studies have shown that APHE was more frequently observed in the ER group than in the non-ER group and was identified as an independent predictor of ER (11, 13). The results of our study were consistent with previous studies. The possible reason may be that APHE was a feature associated with hypervascular progressed HCC and referred to as enhancement of the venous drainage area in the peritumoral liver parenchyma during multistep hepatocarcinogenesis (26). The non-smooth tumor margin has been proven to be closely related to tumor invasion and poor prognosis (16, 27, 28). Ariizumi et al. (29) reported that the incidence of portal vein invasion and intrahepatic metastasis in HCC patients with non-smooth margins was significantly higher than in patients with smooth margins. Additionally, their findings confirmed that the non-smooth margin was an important predictor of ER. In our study, the non-smooth tumor margin was also strongly correlated with ER. As an imaging biomarker with important clinical application value, the non-smooth tumor margin is closely related to tumor heterogeneity and invasive behavior, which leads to a higher probability of ER.

HCC is rare among patients without liver disease, and hepatitis B virus (HBV)-induced cirrhosis is the main risk factor for HCC (30). Yao et al. (31) found that cirrhosis was an independent risk factor associated with postoperative recurrence (p < 0.001). The incidence of cirrhosis in HCC patients with ER was higher than that in patients with late recurrence, but there was no significant statistical difference (p > 0.05). Portolani et al. (32) reported that cirrhosis was significantly associated with ER. Our results also showed that cirrhosis was an independent risk factor for ER. In this study, satellite nodules were defined as nodules that were invisible in images but presented around the primary tumors reported by postoperative pathology, and the presence of satellite nodules significantly predicted ER. In addition, the liver serosal invasion was an independent risk factor for postoperative ER in our study. Few studies have explored the relationship between serosal invasion and ER. Yamamoto et al. (33) reported that serosal invasion was associated with ER (p = 0.031). More studies are needed to confirm this conclusion in the future. Interestingly, in our study, MVI had no significant correlation with ER in the multivariate analysis, though it was a significant factor in the univariate analysis. Numerous studies reported that MVI was a significant risk factor associated with ER of HCC (25, 34–36). The discrepancy existed possibly because MVI was related to the aggressive behavior of the primary tumors. The frequency of MVI in HCC with a diameter less than 5 cm is significantly lower than in large or multifocal HCC as reported in previous studies (37–39). Another possible reason is that the HCC patients with a tumor diameter ≤5 cm generally undergo radical surgical resection, which may have a certain impact on reducing the risk of postoperative ER.

This study has several limitations. Firstly, selection bias was inevitable due to the retrospective nature. In order to increase the reliability, we applied the model obtained from the training set to the validation set. Secondly, our study was a single-center study from areas with a high incidence of HBV or hepatitis C virus infection, so this conclusion may not be applicable to other people with different liver diseases. Thirdly, we developed a prediction model only for ER and did not include late recurrence or long-term survival analyses because of the short postoperative follow-up time, which needs further investigation. Lastly, only patients with a single lesion ≤5 cm were recruited; therefore, this conclusion may not be extended to nodules with a maximum diameter >5 cm or multiple nodules. Thus, the results of this study need to be verified by more extensive and prospective studies in the future.

## Conclusions

In conclusion, our findings showed that the combined model integrated clinical–radiological risk factors with the radiomics signature demonstrated good discriminative ability for predicting ER in HCC patients with a single nodule ≤5 cm, which may serve as a non-invasive and visualized tool in clinical decision-making. More multicenter, prospective studies will be needed to investigate the role of radiomics analysis in clinical practice in the future.

## Data Availability Statement

The raw data supporting the conclusions of this article will be made available by the authors, without undue reservation.

## Ethics Statement

The studies involving human participants were reviewed and approved by the Ethics Committee of Cancer Hospital, Chinese Academy of Medical Sciences. Written informed consent for participation was not required for this study in accordance with the national legislation and the institutional requirements.

## Author Contributions

Study concepts and design: LW, XM, and XZ. Literature research: LW and BF. data collection: LW, BF, ML, and DL. Image analysis: LW and BF. Data analysis: LW, SCW, and SW. Manuscript writing: LW. Manuscript review: XM and XZ. All authors read and approved the final manuscript.

## Funding

This work was sponsored by the PUMC Youth Fund (2017320010).

## Conflict of Interest

Author SCW was employed by GE Healthcare.

The remaining authors declare that the research was conducted in the absence of any commercial or financial relationships that could be construed as a potential conflict of interest.

## Publisher’s Note

All claims expressed in this article are solely those of the authors and do not necessarily represent those of their affiliated organizations, or those of the publisher, the editors and the reviewers. Any product that may be evaluated in this article, or claim that may be made by its manufacturer, is not guaranteed or endorsed by the publisher.

## Abbreviations

AFP, alpha-fetoprotein; AP, arterial phase; APHE, arterial peritumoral hyperenhancement; AUC, area under the curve; DCE, dynamic contrast-enhanced; DP, delayed phase; DWI, diffusion-weighted imaging; ER, early recurrence; HCC, hepatocellular carcinoma; ICC, interclass correlation coefficient; LASSO, least absolute shrinkage and selection operator; MVI, microvascular invasion; NPV, negative predictive value; PPV, positive predictive value; PVP, portal venous phase; ROC, operating characteristic curve; T2WI/FS, fat-suppression.
